# Ultrasound-guided acupotomy release in the treatment of refractory low back pain: A case report

**DOI:** 10.1097/MD.0000000000045046

**Published:** 2025-10-17

**Authors:** Zhanying Tang, Hongkai Zhang, Mingqi Wu, Sidi Zhang

**Affiliations:** a Department of Rehabilitation Medicine, Longhua Hospital Affiliated to Shanghai University of Traditional Chinese Medicine, Shanghai, China.

**Keywords:** acupotomy, minimally invasive procedures, multifidus muscle, musculoskeletal ultrasound, refractory low back pain

## Abstract

**Rationale:**

Chronic refractory low back pain (LBP) poses a significant clinical challenge, characterized by persistent symptoms lasting ≥ 1 year despite extensive conventional treatments (>90 days of pharmacotherapy and physical therapy). The lack of a standardized management strategy necessitates exploration of alternative interventions. Acupotomy, though effective in releasing soft tissue adhesions, is often performed blindly, carrying potential safety risks and limited precision. This case report aims to illustrate the potential of musculoskeletal ultrasound (MSK-US) guidance to enhance the accuracy, safety, and efficacy of acupotomy for this challenging condition.

**Patient concerns:**

A 35-year-old male with a 5-year history of LBP, refractory to prior drug therapy, physiotherapy, and conventional acupuncture, was included.

**Diagnoses:**

Refractory low back pain.

**Interventions:**

He initially underwent a blind acupotomy procedure, releasing tender points in the lumbar 5 to sacral 1 paraspinal region with a 0.8 mm needle. Subsequently, he received 2 sessions of MSK-US-guided acupotomy. This targeted approach utilized real-time, high-frequency ultrasound visualization (in-plane technique) to achieve precise release of the multifidus muscle at the lumbar 5 level, specifically targeting identified myofascial trigger points.

**Outcomes:**

The initial blind acupotomy yielded mild pain reduction (Visual Analog Scale score decreasing from 6–5), though limitations in sitting and standing tolerance persisted. In contrast, the MSK-US-guided acupotomy produced significant relief after the 1st session (Visual Analog Scale decreasing from 5–3), enabling the patient to resume desk work for periods exceeding 30 minutes; near-resolution of symptoms occurred following 2 sessions.

**Lessons:**

This case demonstrates that MSK-US-guided acupotomy facilitates precise tissue release, enabling rapid and significant symptom control in refractory LBP. The technique’s dual mechanism (combining physical adhesion lysis with functional restoration) highlights its potential as a minimally invasive, cost-effective, and superior therapeutic alternative for patients who are not candidates for surgery.

## 1. Introduction

Low back pain (LBP) is characterized by pain, stiffness, and muscular tension localized between the inferior rib margin and gluteal folds.^[1]^ Recognized as a leading global cause of disability (i.e., workforce loss),^[2]^ its etiology encompasses diverse pathologies, including: musculoligamentous sprains/strains, intervertebral disc herniation, and spinal stenosis.^[3]^ Epidemiological studies report a current prevalence of 6.11% to 28.5% in China,^[4]^ aligning with global estimates of 13.1% to 20.3%.^[5]^

1. Clinical burden and therapeutic challenges in refractory LBP

Approximately 10% of chronic LBP cases stem from identifiable pathologies (e.g., vertebral compression fractures, disc herniation) typically requiring surgery. In contrast, the majority present as chronic nonspecific LBP (characterized by mechanical musculoskeletal pain despite normal imaging or nonsurgical disc abnormalities).^[6–8]^

A subset of patients progresses to refractory LBP after inadequate responses to ≥ 90 days of multimodal conservative therapy (e.g., physical therapy, spinal manipulation, opioids/non-opioids, injections, medial branch radiofrequency ablation).^[9–17]^ Current diagnostic criteria define refractory LBP as:

Persistent symptoms ≥ 50% of days within the past year.

Failure of pharmacotherapy plus ≥ 1 physical therapy modality after > 90 days of intervention.^[18]^

Current treatment limitations:

### 1.1. Pharmacotherapy

Non-steroidal anti-inflammatory drugs (oral/topical) are first-line but offer limited efficacy (moderate evidence) and carry gastrointestinal/neurological risks.^[19]^

Topical non-steroidal anti-inflammatory drugs demonstrate superior safety versus oral formulations.^[20]^

### 1.2. Non-pharmacological interventions

Supervised exercise (e.g., Pilates, McKenzie therapy) shows modest benefits for pain/function.^[21–23]^

Behavioral therapies exhibit comparable efficacy across subtypes (response/operant/cognitive).^[23]^

Insufficient evidence supports spinal manipulation,^[15]^ massage,^[24]^ yoga,^[25]^ or multidisciplinary rehabilitation.^[26]^

### 1.3. Physical modalities

Electrostimulation,^[27]^ therapeutic ultrasound,^[28,29]^ and microwave therapy lack robust randomized controlled trial (RCT) evidence, limiting their use to adjunctive roles.^[27,30,31]^

### 1.4. Pathogenesis-driven approaches

Multifidus muscle dysfunction (inducing segmental instability) is a key pathomechanism.^[18,32]^ Although implantable neurostimulators attempt neuromuscular restoration, inconclusive benefits and high complication rates (e.g., surgical adverse events) persist.^[18]^ No evidence-based gold standard currently exists for refractory LBP management.

1. Multifidus muscle dysfunction: a core pathological mechanism in refractory LBP.

Normal physiological lumbar function depends on spinal stability,^[33]^ and impairment of this stability is closely linked to LBP. The multifidus muscle, situated innermost along the spinal column with its most developed portion in the lumbosacral region, constitutes a critical component of the active contraction control system. Its functional morphology is intimately associated with lumbar stability. Previous studies indicate that the lumbar multifidus contributes up to two-thirds of segmental stability at the Lumbar 4 to 5 (L4/5) level.^[34]^ Using musculoskeletal ultrasound (MSK-US), Zhang et al demonstrated that patients with chronic LBP exhibit significant atrophy of the deep lumbar multifidus muscle. Compared to healthy individuals, these patients show reduced resting muscle thickness and cross-sectional area. Ultrasonography further reveals characteristic alterations:

Linear or punctate hyperechoic foci.

Heterogeneous hypoechoic areas.

Blurred feather-like, reticular, or absent intramuscular septa.

These findings indicate muscle atrophy, fatty infiltration, and scar-like fibrotic changes within the multifidus of chronic LBP patients.^[35]^

1. Ultrasound-guided acupotomy for multifidus release in refractory LBP

Acupotomy combines acupuncture needles with surgical blades to unblock meridians, promote qi-blood circulation^[36,37]^ and alleviate pain. Its high efficiency, cost-effectiveness, convenience, and short treatment course make it a therapy promoted by China’s National Administration of Traditional Chinese Medicine.^[38]^ Previous studies confirm its efficacy in chronic LBP.^[39]^

However, traditional acupotomy carries safety risks due to blind procedural nature. Clinically, operators cannot visualize subcutaneous anatomy, relying solely on palpation and experience. This leads to frequent adverse events and complications, limiting its application in refractory LBP.

Recent advancements in MSK-US offer solutions.

High-frequency ultrasound enables noninvasive, real-time dynamic assessment of musculoskeletal disorders (e.g., strains, space-occupying lesions, inflammatory/atrophic diseases)

Operator- and position-independent reproducibility.

Integrating MSK-US guidance transforms acupotomy into a visualized procedure that:

Pinpoints needle entry sites/depth.

Prevents nerve/vascular injury.

Allows real-time lesion assessment and technique adjustment.

Reduces intraoperative bleeding and needle-induced pain.

Minimizes patient anxiety, enhancing compliance and trust.

Yang et al^[40]^demonstrated that ultrasound-guided acupotomy provides superior pain relief and functional improvement in chronic nonspecific LBP compared to conventional acupotomy.

## 2. Key mechanisms^[37]^

1. Mechanical adhesion release (surgical blade effect).
2. Improved microcirculation (restored blood flow/peripheral nerve decompression).
3. Neuromodulation (multifidus muscle motor function recovery, addressing instability-related pain).
4. Anti-inflammatory effects (reduced local inflammatory mediators).

## 3. Patient consent statement

Written informed consent has been obtained from the patient. After fully understanding the purpose, process, potential risks, and benefits of this study, the patient voluntarily signed the informed consent form on August 16, 2024, agreeing to the use of their personal information, medical condition data, treatment process and treatment results, etc for the analysis, writing of this study and its publication. This study strictly adheres to relevant ethical guidelines, and has implemented strict confidentiality measures for the patient’s privacy information to ensure that the patient’s personal privacy is not violated.

## 4. Clinical application example: ultrasound-guided acupotomy for refractory LBP (Table 1)

### 4.1. Patient profile

Male, 35 years, height 170 cm, weight 60 kg, body mass index 20 kg/m². Presented to the Rehabilitation Department of Longhua Hospital Affiliated to Shanghai University of Traditional Chinese Medicine with a 5-year history of recurrent LBP.

**Table 1:** Event timeline.

Event sequence	Event date
The patient visited the department of rehabilitation medicine with a chief complaint of recurrent low back pain for more than 5 years	August 16, 2024
The patient was asked about symptoms and underwent a clinical physical examination in addition to radiology tests, which revealed no obvious abnormalities. The patient was diagnosed with “chronic low back pain.”	August 16, 2024
First visit, conventional needle-knife therapy is used.	August 16, 2024
Follow-up visit. The patient reported that the low back pain was better than before, but the effect was not significant. Planned to perform needle knife release treatment under ultrasound guidance.	August 30, 2024
Third visit. The patient reported that the low back pain was significantly improved compared with before, the last needle-knife treatment under ultrasound guidance.	September 13, 2024

### 4.2. Clinical history

Initial injury occurred during basketball with direct lumbar impact, causing immediate pain and restricted movement. Symptoms partially resolved with rest but recurred persistently over 5 years. Pain characteristics:

Dull ache localized to impact site.

Aggravated by prolonged sitting/standing.

Previous treatments included oral medications, massage, acupuncture, physiotherapy, traction, and herbal fumigation with minimal relief. Current pain severity interferes with work and daily activities.

### 4.3. Physical examination

Tenderness over lumbar paraspinal muscles.

Lumbar flexion/extension/lateral bending: normal.

Straight leg raise test: negative bilaterally.

Patrick test: negative.

Lumbar percussion pain: negative.

L4-L5/lumbar 5 to sacral 1 paraspinal tenderness: positive.

Lumbar 3 transverse process tenderness: negative.

No sensory abnormalities in lower extremities.

### 4.4. Diagnostic imaging

**Figure 1.**
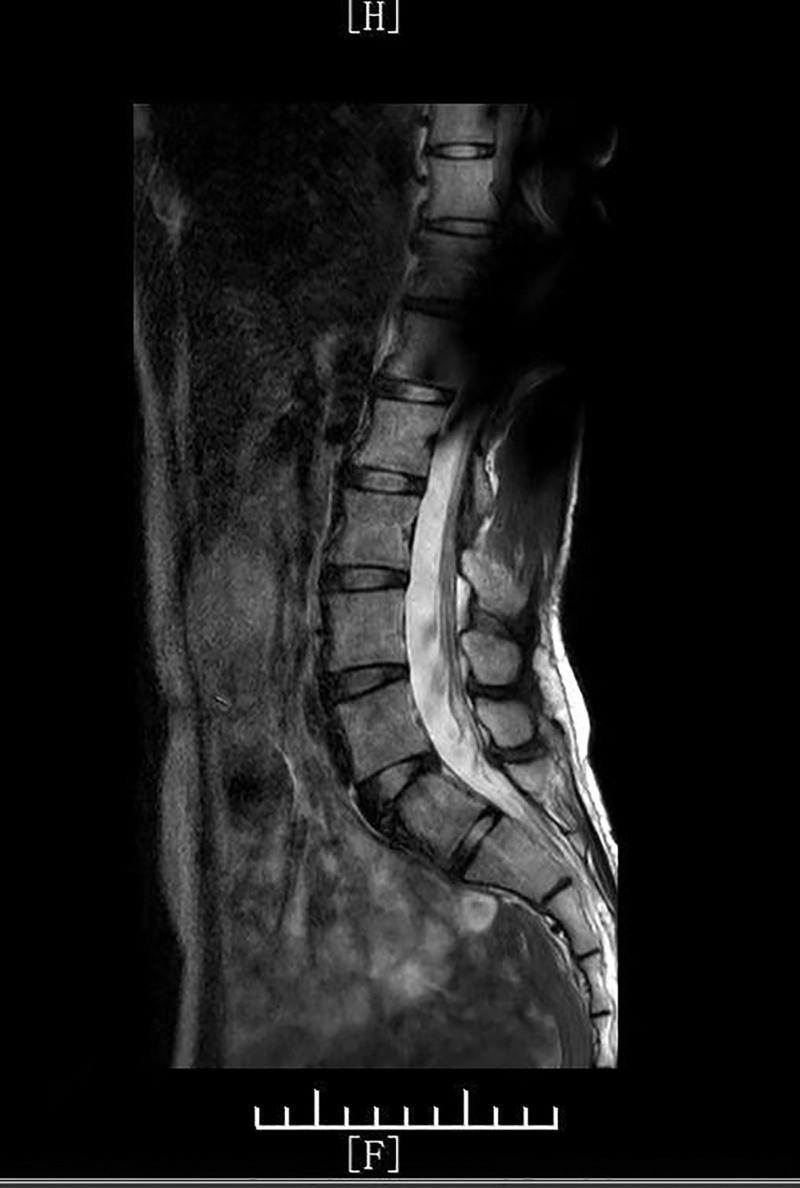
The density of the lumbar and sacral vertebral bodies is normal on the MRI, the L4–L5 intervertebral disc is slightly degenerated, the lumbar canal is not narrowed, and no other obvious abnormalities are seen. L4–5 = lumbar 4 to 5, MRI = magnetic resonance imaging.

Lumbar magnetic resonance imaging: Mild L4-L5 disc degeneration without spinal canal stenosis (Fig. 1).

### 4.5. Baseline assessment

Visual Analog Scale (VAS): 7/10.

Oswestry Disability Index (ODI): 35/50(Table 2).

**Table 2:** VAS and ODI scores at the 1st visit.

1. VAS pain scoring criteria (0–10 points)						
---- ---- ---- ---- ---- ---- ---- ---- ---- ---- 0 1 2 3 4 5 6 7 8 9 10						
Explanation: 0 means no pain, 10 means the most severe pain. 0 points: no pain. Below 3 points: there is mild pain and it is bearable. 4–6 points: the patient has pain and it affects sleep, but it is still bearable. 7–10 points: the patient has relatively severe pain, the pain is unbearable, it affects appetite and sleep.						
2. ODI scoring (0–5 points pain progression, 0 points means completely no pain, 5 points means extreme pain and the most severe degree of disability)						
	0	1	2	3	4	5
Pain intensity					√	
Daily life self-care (washing, dressing, etc)				√		
Lifting capacity					√	
Walking				√		
Sitting					√	
Standing					√	
Sleep quality					√	
Sexual life						√
Social activities and travel (outing)					√	
Total score: 35	Filling – in time: August 16, 2024					

ODI = Oswestry Disability Index, VAS = Visual Analog Scale.

No lower extremity radiculopathy.

Normal bowel/bladder function.

Diagnosis

Refractory LBP.

Therapeutic intervention

First treatment

Conventional acupotomy at 4 tender points (L4–L5/lumbar 5 to sacral 1 paraspinal regions).

Procedure:

Local skin sterilization.

0.5% lidocaine infiltration anesthesia.

Release with 0.8 × 80 mm acupotomy needle.

Compression hemostasis and sterile dressing.

Second treatment (2-week follow-up).

VAS: 6/10.

ODI: 29/50 (Table 3).

**Table 3:** VAS and ODI scores at the 2nd visit.

1. VAS pain scoring criteria (0–10 points)						
---- ---- ---- ---- ---- ---- ---- ---- ---- ---- 0 1 2 3 4 5 6 7 8 9 10						
Explanation: 0 means no pain, 10 means the most severe pain. 0 points: no pain. Below 3 points: there is mild pain and it is bearable. 4–6 points: the patient has pain and it affects sleep, but it is still bearable. 7–10 points: the patient has relatively severe pain, the pain is unbearable, it affects appetite and sleep.						
2. ODI scoring (0–5 points pain progression, 0 points means completely no pain, 5 points means extreme pain and the most severe degree of disability)						
	0	1	2	3	4	5
Pain intensity				√		
Daily life self-care (washing, dressing, etc)				√		
Lifting capacity					√	
Walking				√		
Sitting				√		
Standing				√		
Sleep quality				√		
Sexual life					√	
Social activities and travel (outing)				√		
Total score: 29	Filling – in time: August 30, 2024					

ODI = Oswestry Disability Index, VAS = Visual Analog Scale.

Suboptimal pain relief (persistent nocturnal stiffness affecting sleep).

Ultrasound-guided multifidus release:

Identical tender point localization.

Sterilization and local anesthesia.

Application of sterile ultrasound gel.

Transverse placement of high-frequency probe over L5 transverse process.

**Figure 2.**
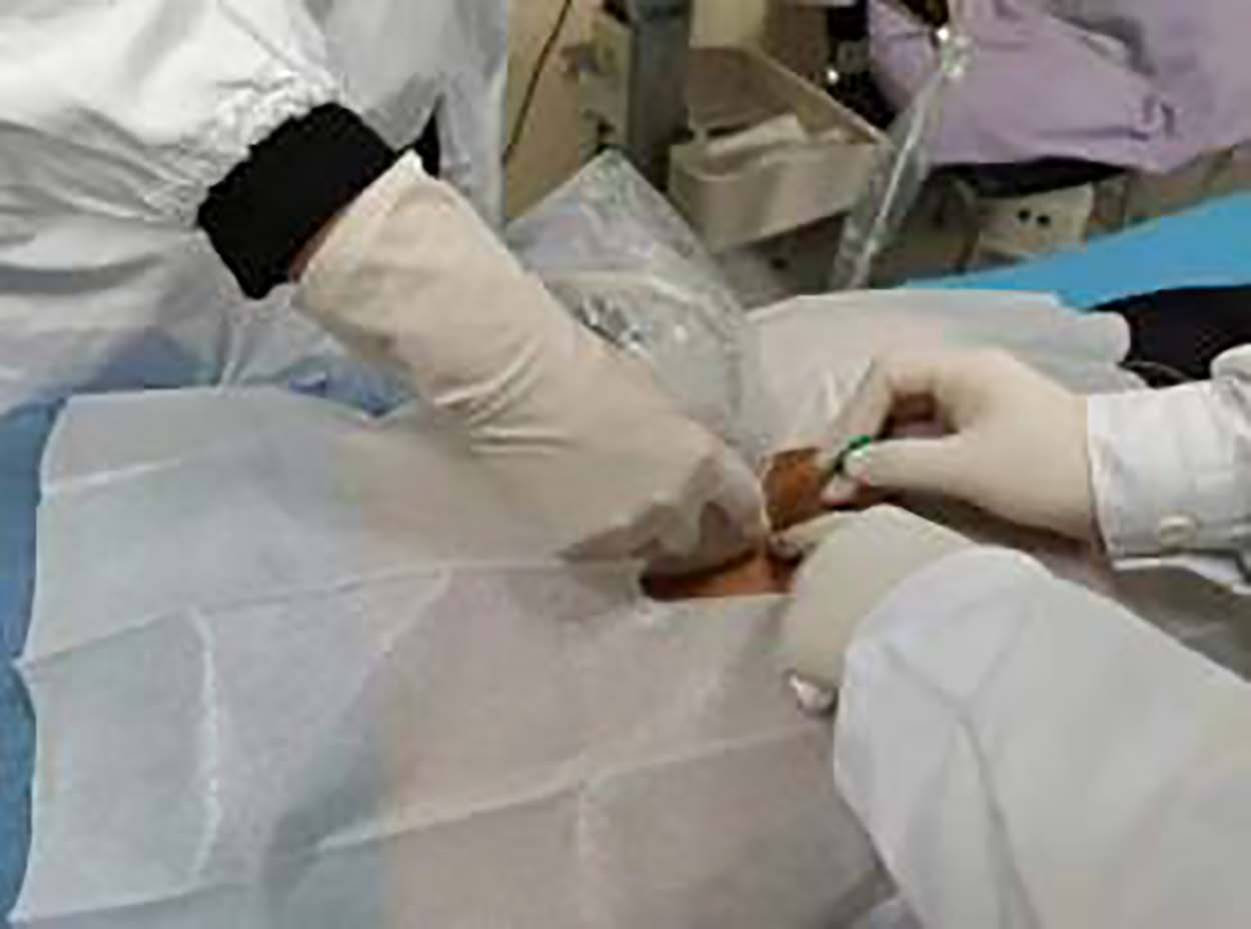
Ultrasound-guided trigger point needling.

**Figure 3.**
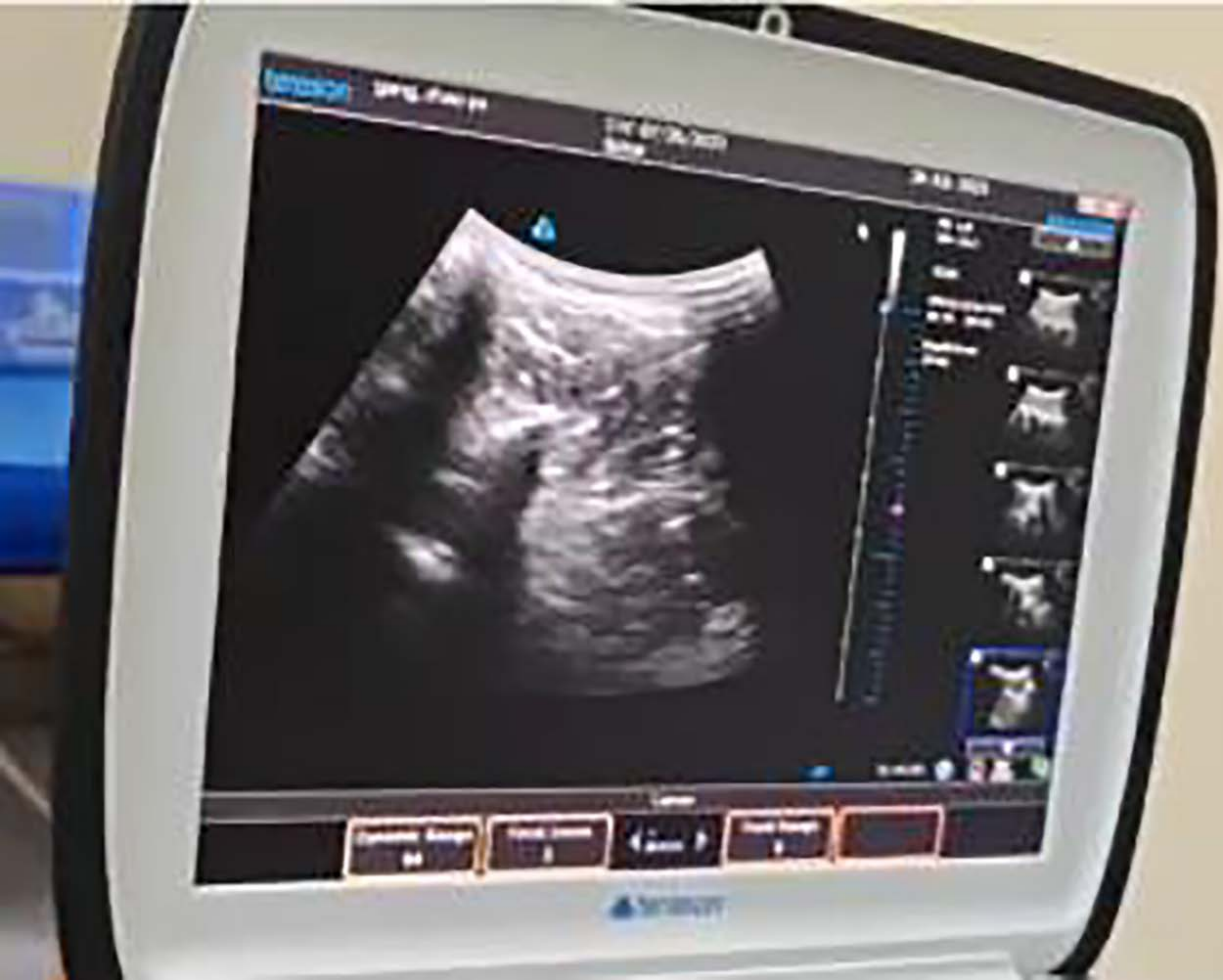
Ultrasound-guided trigger point needling.

Real-time in-plane needle insertion (0.8 × 80 mm acupotomy) (Figs. 2 and 3).

Sustained release at identified trigger points.

Hemostasis and sterile dressing.

Third treatment (2-week interval).

VAS: 3/10.

ODI: 22/50 (Table 4).

**Table 4:** VAS and ODI scores at the 3rd visit.

1. VAS Pain Scoring Criteria (0–10 points)						
---- ---- ---- ---- ---- ---- ---- ---- ---- ---- 0 1 2 3 4 5 6 7 8 9 10						
Explanation: 0 means no pain, 10 means the most severe pain. 0 points: no pain. Below 3 points: there is mild pain and it is bearable. 4–6 points: the patient has pain and it affects sleep, but it is still bearable. 7–10 points: the patient has relatively severe pain, the pain is unbearable, it affects appetite and sleep.						
2. ODI scoring (0–5 points pain progression, 0 points means completely no pain, 5 points means extreme pain and the most severe degree of disability)						
	0	1	2	3	4	5
Pain intensity			√			
Daily life self-care (washing, dressing, etc)			√			
Lifting capacity				√		
Walking			√			
Sitting			√			
Standing				√		
Sleep quality			√			
Sexual life				√		
Social activities and TRAVEL (outing)				√		
Total score: 22	Filling – in time: September 13, 2024					

ODI = Oswestry Disability Index, VAS = Visual Analog Scale.

Two additional ultrasound-guided procedures achieved complete resolution of symptoms (Table 5).

**Table 5:** VAS and ODI scores at the 4th visit.

1. VAS pain scoring criteria (0–10 points)						
---- ---- ---- ---- ---- ---- ---- ---- ---- ---- 0 1 2 3 4 5 6 7 8 9 10						
Explanation: 0 means no pain, 10 means the most severe pain. 0 points: no pain. Below 3 points: there is mild pain and it is bearable. 4–6 points: the patient has pain and it affects sleep, but it is still bearable. 7–10 points: the patient has relatively severe pain, the pain is unbearable, it affects appetite and sleep.						
2. ODI scoring (0–5 points pain progression, 0 points means completely no pain, 5 points means extreme pain and the most severe degree of disability)						
	0	1	2	3	4	5
Pain intensity		√				
Daily life self-care (washing, dressing, etc)		√				
Lifting capacity		√				
Walking		√				
Sitting		√				
Standing		√				
Sleep quality		√				
Sexual life			√			
Social activities and travel (outing)		√				
Total score: 10	Filling – in time: September 27, 2024					

ODI = Oswestry Disability Index, VAS = Visual Analog Scale.

## 5. Detailed outcomes report

The therapeutic outcomes, rigorously assessed using validated instruments at each intervention stage, are detailed below:

1. Pain intensity (VAS):

Baseline: severe pain, VAS score = 7/10.

Post-blind acupotomy (2-week follow-up): modest improvement observed. VAS score decreased to 6/10.

Post-first ultrasound-guided acupotomy (2-week follow-up): significant pain reduction achieved. VAS score decreased markedly to 3/10.

Post-second ultrasound-guided acupotomy (final follow-up): near-complete resolution of pain. VAS score stabilized at 3/10 (indicating mild residual discomfort).

Summary: overall VAS reduction from baseline to final follow-up was 57% (7→3). The incremental reduction attributable specifically to the 1st US-guided session was substantial (VAS 6→3, 50% decrease).

1. Functional disability (ODI):

Baseline: significant functional impairment, ODI score = 35/50 (70% disability).

Post-blind acupotomy (2-week follow-up): modest functional improvement. ODI score decreased to 29/50 (58% disability), representing a 17% reduction from baseline.

Post-first ultrasound-guided acupotomy (2-week follow-up): clinically meaningful functional recovery. ODI score decreased to 22/50 (44% disability), a 37% reduction from baseline and a 24% reduction from the post-blind intervention state.

Final status: disability reduced to a mild-moderate level (ODI 22/50).

Summary: overall ODI reduction from baseline was 37% (35→22). The most significant functional gains coincided with the US-guided interventions.

1. Key functional milestones:

Baseline: severe limitations in sitting and standing tolerance, preventing sustained desk work.

Post-blind acupotomy: no significant improvement in sitting/standing tolerance or work capacity reported.

Post-first ultrasound-guided acupotomy: dramatic improvement in functional capacity. The patient regained the ability to sit for periods exceeding 30 minutes, enabling a partial return to desk-based occupational activities previously impossible.

Post-second ultrasound-guided acupotomy: sustained functional improvement, consolidating the gains from the 1st US-guided session. Near-normalization of daily activities was reported.

1. Symptom resolution

The persistent nocturnal lumbar stiffness, which significantly disrupted sleep prior to the US-guided interventions, was completely resolved following the two ultrasound-guided acupotomy sessions.

Conclusion of outcomes: the transition from landmark-guided to MSK-US-guided acupotomy, specifically targeting the dysfunctional multifidus muscle under real-time visualization, was associated with a profound and clinically significant improvement in both pain intensity and functional disability. The most substantial and rapid gains occurred precisely after the implementation of ultrasound guidance, leading to the restoration of key functional abilities (sitting tolerance > 30 mins) and resolution of sleep-disrupting symptoms. The cumulative ODI reduction of 37% surpasses the commonly accepted minimal clinically important difference (MCID) for ODI (estimated at 10–30% depending on baseline severity), underscoring the clinical relevance of the observed improvements.

## 6. Discussion and perspectives

This report details a case of chronic LBP refractory to conventional therapies, including acupuncture and physiotherapy, satisfying criteria for refractory LBP. Initial symptom management employing landmark-guided acupotomy yielded modest yet discernible improvement from baseline. However, a marked clinical response, culminating in near-complete resolution of symptoms, was achieved only after implementing ultrasound-guided acupotomy specifically targeting the multifidus muscle.

These findings corroborate previous observations by Yang Dan et al^[40]^ regarding the efficacy of ultrasound-guided acupotomy in chronic nonspecific LBP. Nevertheless, critical distinctions between the present case and the cohort described by Yang Dan merit emphasis, potentially expanding the application scope of this technique. Firstly, the interventional target differed significantly: the current report focused on the deep multifidus muscle, whereas Yang Dan et al targeted the more superficial erector spinae. Secondly, the chronicity and treatment resistance were more pronounced, involving a 5-year refractory course compared to patients with symptoms of less than 1 year. Thirdly, the pathological complexity and severity appeared greater, characterized by deep-seated muscular pathology unresponsive to prior interventions. This heightened disease burden inherently complicates effective therapeutic planning and execution.

This case, therefore, underscores the specific clinical utility of MSK-US-guided acupotomy precisely targeting the multifidus muscle in the context of refractory LBP. It addresses a significant gap in the literature concerning effective interventional strategies for patients with deep myofascial pathologies contributing to longstanding, treatment-resistant pain. While evidence supports various approaches for chronic LBP, robust data on optimized, minimally invasive techniques specifically for refractory presentations involving deep stabilizers like the multifidus remain limited.

Clinical implications: persistent LBP represents a substantial global health burden with suboptimal control rates under prevailing orthodox and traditional therapeutic paradigms. MSK-US-guided acupotomy emerges as a viable and potentially superior strategy for selected refractory cases. Its primary advantages include enhanced anatomical precision enabling accurate targeting of deep structures, minimized iatrogenic tissue trauma translating to improved patient tolerance and faster recovery, and favorable cost-effectiveness relative to more invasive surgical interventions or prolonged, ineffective conservative management.

Study limitations: while this case report demonstrates the potential benefits of MSK-US-guided acupotomy for refractory LBP, several limitations must be acknowledged to provide a balanced interpretation of the findings: inherent limitations of a case report design: as a report of a single case, the findings are not generalizable to the broader population. The outcomes observed in this individual may not be replicable in others due to variations in anatomy, pain pathophysiology, and individual response to treatment. The positive results require validation through large-scale RCTs; lack of blinding and control: the nature of the intervention makes blinding of the practitioner and patient impossible. Furthermore, the sequential treatment design (blind acupotomy followed by US-guided acupotomy) lacks a parallel control group. Therefore, the observed improvement cannot be definitively disentangled from potential placebo effects, natural history of the condition, or the cumulative effect of multiple procedures; operator dependence: the success of MSK-US-guided interventions is highly dependent on the operator’s skill and expertise in both MSK-US and acupotomy techniques. The proficiency required may limit the immediate widespread applicability of this technique in all clinical settings; short-term follow-up: this report primarily describes short-term outcomes immediately following the intervention. The long-term durability of the symptomatic relief and functional improvement achieved with US-guided acupotomy remains unknown and warrants investigation with extended follow-up periods; standardization of protocol: while the in-plane technique was used, the precise parameters of the “release” (e.g., number of needle passes, extent of soft tissue dissection) are based on real-time sonographic findings and operator judgment, which are difficult to standardize completely. Future studies should aim to develop a more standardized protocol for reproducibility.

By acknowledging these limitations, we aim to present a transparent view of our research. Despite these constraints, this case offers valuable preliminary evidence and serves as a catalyst for future rigorous studies to confirm the efficacy and optimize the protocol for US-guided acupotomy in managing refractory LBP.

Future directions: to consolidate these promising findings and address existing gaps, further investigation is warranted. Priority should be placed on: systematic validation of the therapeutic efficacy and durability of MSK-US-guided multifidus acupotomy through adequately powered RCTs comparing it to standard care, sham procedures, and other guided techniques; comprehensive establishment of its short- and long-term safety profile, including the incidence of procedure-related adverse events; and elucidation of the underlying mechanistic pathways contributing to its clinical effects, particularly regarding neuromodulation, microcirculatory changes, and anti-inflammatory actions, utilizing advanced imaging and biomarker analyses.

In summary, this case highlights ultrasound-guided acupotomy targeting the multifidus muscle for refractory LBP. The patient exhibited higher disease burden with deep-seated pathology compared to typical chronic LBP cases, rendering effective therapeutic planning considerably more challenging. This underscores its clinical implications for managing refractory presentations.

## Author contributions

**Conceptualization:** Hongkai Zhang, Zhanying Tang.

**Data curation:** Hongkai Zhang, Zhanying Tang.

**Formal analysis:** Hongkai Zhang, Zhanying Tang.

**Funding acquisition:** Zhanying Tang.

**Investigation:** Mingqi Wu, Sidi Zhang.

**Methodology:** Zhanying Tang.

**Project administration:** Hongkai Zhang.

**Resources:** Hongkai Zhang.

**Software:** Mingqi Wu, Sidi Zhang.

**Supervision:** Mingqi Wu, Zhanying Tang.

**Visualization:** Sidi Zhang.

**Writing – original draft:** Hongkai Zhang.

**Writing – review & editing:** Zhanying Tang.
